# Mechanism of Rescue of Na^+^,K^+^-ATPase Function in Neurological Disease Revealed by New Super-Efficient Second-Site Mutations

**DOI:** 10.3390/ijms27188424

**Published:** 2026-09-21

**Authors:** Rikke Holm, Mads S. Toustrup-Jensen, Hang N. Nielsen, Jens Peter Andersen, Bente Vilsen

**Affiliations:** Department of Biomedicine, Aarhus University, DK-8000 Aarhus, Denmark; mtj@biomed.au.dk (M.S.T.-J.); hangnielsen@gmail.com (H.N.N.); jpa@biomed.au.dk (J.P.A.)

**Keywords:** Na^+^,K^+^-pump, P-type ATPase, neurological disorder, disease mutation, rescue mutation, Na^+^ affinity, COS-1 cell expression system

## Abstract

Serious neurological disorders are caused by mutation of Na^+^,K^+^-ATPase α1–α3 isoforms. Several disease mutations reduce the affinity for Na^+^, which in vivo results in an increase in the intracellular Na^+^ concentration with a secondary rise in intracellular Ca^2+^. Current treatments are ineffective, as they do not address Na^+^,K^+^-ATPase function. Hence, a platform for the development of a rational therapeutic approach that restores Na^+^ affinity is needed. Using the COS-1 cell expression system, one of the most conspicuous reductions in Na^+^ affinity of Na^+^,K^+^-ATPase has been reported for the neurological disease mutation α3-D923N of the α3 isoform. We describe here second-site mutations (“rescue mutations”) that fully restore Na^+^ affinity of α3-D923N and the corresponding rat α1-D928N and reveal the underlying molecular mechanism. Paradoxically, this insight evolved from investigation of a rapid-onset ataxia Na^+^,K^+^-ATPase mutant, α3-G316S, that exhibits an increased affinity for Na^+^. When applied as second-site mutations, α3-G316S and the corresponding rat α1-G321S are super-efficient rescuers of the reduced Na^+^ affinity of α3-D923N and rat α1-D928N. Replacement of the glycine with serine leads to a clash between transmembrane helices in the K^+^-bound E_2_ state of Na^+^,K^+^-ATPase but not in the Na^+^-bound E_1_ state. A similarly strong rescuing power is observed for second-site mutations breaking a salt bridge present only in the E_2_ state. The rescue is due to a structural change resembling the rearrangement of the ion binding sites occurring during the normal Na^+^,K^+^-ATPase transport cycle as part of the E_2_-to-E_1_ conformational transition.

## 1. Introduction

**Figure 1:**
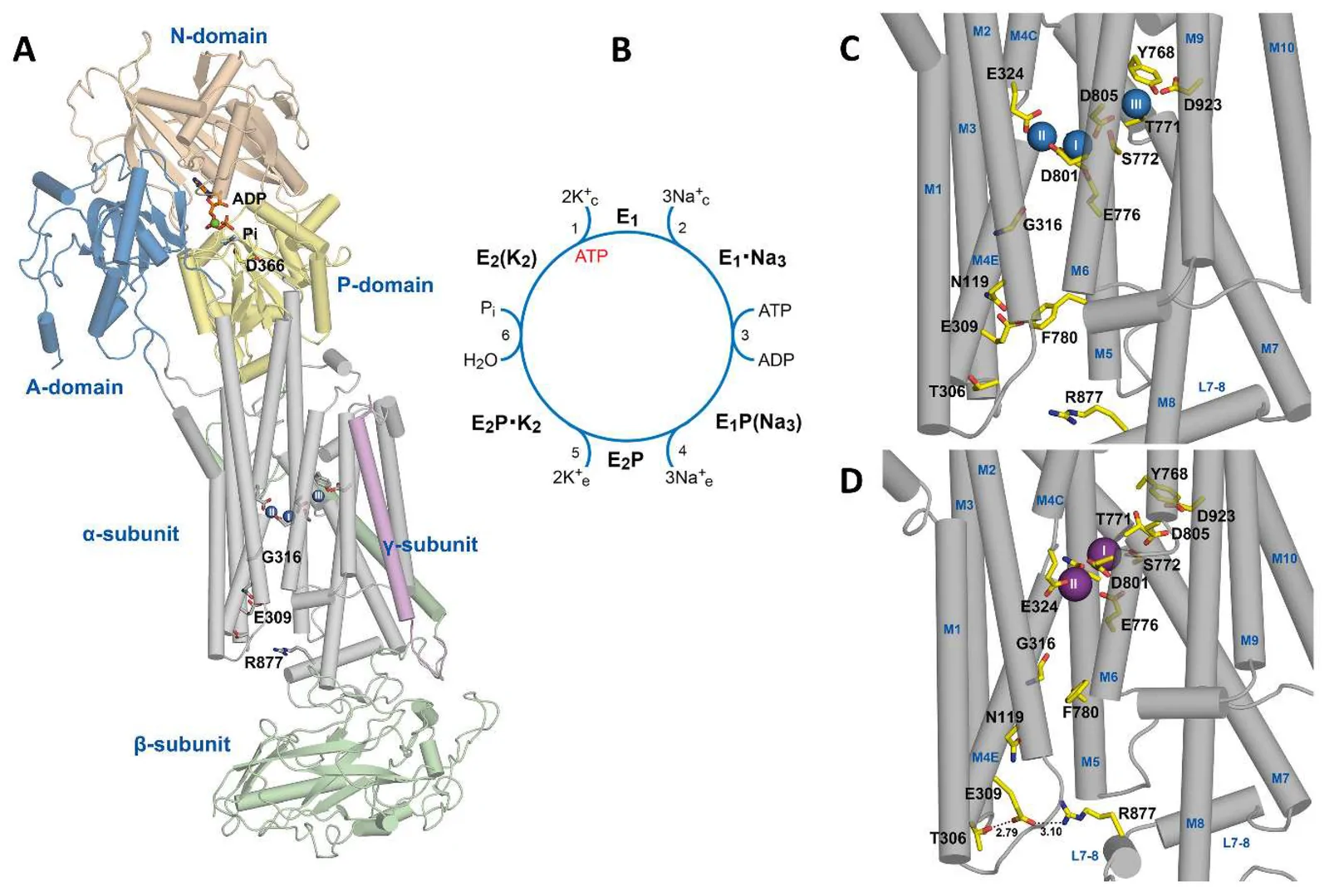
Na^+^,K^+^-ATPase structural elements in focus in the present study and transport cycle. (**A**) Overall structure of the Na^+^,K^+^-ATPase in the Na^+^-bound E_1_ state (E_1_P(Na_3_) form, PDB ID: 3WGV [5]). The cytoplasmic side is upward and the extracellular side is downward. The P-, N-, and A-domains of the cytoplasmic headpiece of the α subunit are colored yellow, sandy, and blue, respectively. The transmembrane part of the α subunit consisting mainly of ten helices shown as cylinders is colored gray, the β subunit green, and the γ subunit (FXYD protein) violet. Labels indicate ADP in the catalytic site with AlF_4_ mimicking P_i_ in transition state, the aspartic acid residue D366 receiving the phosphate, the three bound Na^+^ (blue spheres numbered I, II and III according to accepted nomenclature [5]) and the three residues E309, G316 and R877 (α3 numbering) playing central roles in the rescue of Na^+^ affinity described here. (**B**) Na^+^,K^+^-ATPase transport cycle [2,3,4] with partial reactions numbered 1–6. There are four main conformational states: E_1_ has open cytoplasmic-facing ion-binding sites with preference for Na^+^, E_1_P has Na^+^ bound in an occluded state (i.e., with no access to medium on either side of the membrane), indicated by parentheses, E_2_P has open extracellular-facing ion-binding sites with preference for K^+^, E_2_ has K^+^ bound in an occluded state. P indicates phosphorylation of the aspartate in the catalytic site. Free Na^+^ and K^+^ ions in the medium are labeled c and e for cytoplasmic and extracellular side, respectively. The red ATP indicates ATP bound in a non-phosphorylating allosteric mode, enhancing the rate of the K^+^-deoccluding E_2_-to-E_1_ conformational transition. (**C**) Zoomed-in view of the transmembrane region of the Na^+^-bound E_1_ state in (**A**). Most residues mentioned in the text as well as some crucial ion-binding residues are highlighted by stick representation with oxygen atoms red, nitrogen atoms blue and carbon atoms yellow and are labeled according to α3 numbering (five lower than the rat α1 also examined in the present study). The transmembrane helices (M1–M10, numbered from the N-terminus) are labeled with small blue letters (M4 subdivided in two parts separated by the non-helical ion-binding region: M4E closest to the extracellular side and M4C closest to the cytoplasmic side). (**D**) Similar presentation of the transmembrane region of the E_2_ state, which has two bound K^+^ (purple spheres labeled I and II, E_2_∙P_i_(K_2_) form, PDB ID 2ZXE [6] with a threonine inserted in place of serine at position T306 by modeling to mimic the human α1 and α3 isoforms). Dotted lines indicate potential hydrogen bond and salt bridge with lengths given in Å.

Na^+^,K^+^-ATPase (a.k.a. Na^+^,K^+^-pump) is a membrane-bound enzyme responsible for creating vital gradients for Na^+^ and K^+^ across the plasma membrane in all animal cells by exchanging three intracellular Na^+^ for two extracellular K^+^ in each ATP hydrolysis cycle [1,2,3,4,5,6,7]. The Na^+^,K^+^-ATPase protein consists of a catalytic α subunit responsible for the transport of Na^+^ and K^+^, a β subunit assisting in the folding and translocation of the α subunit to the plasma membrane, as well as, in most cases, a regulatory FXYD-subunit not obligatory for the Na^+^ and K^+^ transport activity of the enzyme. The cytoplasmic headpiece of the α subunit is separated into three domains, P, N and A, which all contribute residues to the catalytic ATP hydrolysis site. The P- and A-domains are linked to a transmembrane sector consisting of ten mostly helical segments (M1–M10) of which M4, M5, M6 and M8 donate residues to the binding of the transported ions [5,6,7] (Figure 1). The α subunit is encoded by four different genes, resulting in four highly homologous isoforms, α1–α4, that are expressed and regulated differentially [8]. The α1 isoform is ubiquitously expressed, i.e., in most tissues including the nervous system, whereas α2 is expressed specifically in glial cells of the nervous system as well as in heart and skeletal muscle, α3 is expressed predominantly in neurons, and α4 is expressed exclusively in testis [8,9].

Beginning with the discovery of Na^+^,K^+^-ATPase α2 mutations causing hemiplegic migraine [10] and α3 mutations causing rapid-onset dystonia parkinsonism (RDP) [11], alternating hemiplegia of childhood (AHC) [12], or CAPOS (cerebellar ataxia, areflexia, pes cavus, optic atrophy, and sensorineural hearing loss) syndrome [13], multiple neurological disorders are now known to be associated with mutation of the Na^+^,K^+^-ATPase α1–α3 isoforms [14]. It is well established that α3 variants form a continuum of neurological phenotypes with partially overlapping clinical features [15,16,17,18]. Pathogenic α1 variants were first reported for somatic mutations giving rise to aldosterone-producing adenomas (APAs) [19,20]. Germline α1 mutations were later reported to cause Charcot–Marie–Tooth disease, which affects motor neurons and sensory neurons of peripheral nerves [21]. However, soon thereafter the clinical spectrum of α1 disorders was further expanded with a disease phenotype consisting of renal magnesium wasting accompanied by seizures (persisting despite magnesium supplementation) and cognitive delay, thus demonstrating the involvement of both the kidneys and the central nervous system [22]. The α1 disorders are now known to include several predominantly neurological phenotypes [23,24,25,26].

The specific pathophysiological mechanisms underlying the various Na^+^,K^+^-ATPase disease phenotypes are enigmatic, although the phenotypic traits in one way or another are attributable to mutation of one of the two alleles encoding a specific isoform of Na^+^,K^+^-ATPase. Haploinsufficiency resulting from reduced Na^+^,K^+^-pump activity may contribute in many cases, caused by a reduced expression level or functional defect of the α subunit encoded by the affected allele (including reduced affinity for Na^+^ and/or K^+^ or slowing or complete block of an essential conformational change in the transport cycle). Reduced Na^+^,K^+^-pump activity leads to an increase in the intracellular Na^+^ concentration and decrease in the intracellular K^+^ concentration, as observed in cultured cells expressing pathogenic Na^+^,K^+^-ATPase mutants [27]. The increase in intracellular Na^+^ reduces the activity of various transporters driven by the Na^+^ gradient, including, in neurons, neurotransmitter re-uptake and Na^+^/Ca^2+^ exchange, one effect being an increase in the intracellular Ca^2+^ concentration with a resulting increase in Ca^2+^ signaling and neurotransmitter release. The reduced cellular K^+^ uptake in neurons with dysfunctional Na^+^,K^+^-ATPase, and consequent decrease in the K^+^ gradient across the membrane, may also contribute to increase excitability. Such effects are likely to be translated differently to functional impact in the various neuronal cell types and even in various parts of a neuron (e.g., inhibitory versus excitatory neuron, axon versus dendrites; for a more detailed account, see [14] and Section 11 of [16]).

A leak of Na^+^ through the mutant Na^+^,K^+^-ATPase protein into the cell has been implicated as a mechanism causing dominant negative effects (“gain of toxic function”). Although the observed Na^+^ leaks through pathogenic Na^+^,K^+^-ATPase mutants generally appear too small to depolarize the membrane potential substantially, the leaks may contribute to pathogenicity through a reduction in the net outflux of Na^+^, thus further exacerbating the rise in intracellular Na^+^ caused by a reduced pumping rate [20,28,29].

At the cellular level, mechanisms that might cause dominant negative effects include protein misfolding with alteration of Na^+^,K^+^-ATPase membrane distribution (e.g., ER retention) and activation of the unfolded protein response leading to apoptosis, as well as allelic competition between α subunits for interaction with the β subunit or another critical chaperone [30,31,32].

More than 200 pathogenic variants of the Na^+^,K^+^-ATPase gene family have been reported [14]. Presently, treatments of disorders associated with Na^+^,K^+^-ATPase mutations are rather ineffective, ameliorating or dampening acute symptoms to various extents, e.g., seizures with antiepileptic drugs and sedatives, or reducing the frequency and intensity of plegic attacks with Ca^2+^- and Na^+^-channel blockers such as flunarizine, without curing the patients [18]. Presently, the surgical removal of aldosterone-producing adenomas constitutes the only example of an available treatment eliminating the underlying cause of the symptoms [19,20]. While successful gene editing to correct Na^+^,K^+^-ATPase mutations has recently been performed in mouse models of AHC, this promising approach must await further development, before it becomes available for treatment of human Na^+^,K^+^-ATPase disease [33]. In those cases where the function of Na^+^,K^+^-ATPase is compromised by a disease mutation, a logical therapeutic strategy would be to try to rescue the function at the protein level. Hence, there is a need for an agonist that stimulates the transport activity of Na^+^,K^+^-ATPase disease mutants, analogously to the recently described agonistic effect of polyoxotungstates on purinergic P2 receptors [34]. Several of the α1- and α3-mutations associated with severe disease phenotypes are located in the membrane domain in or near the ion binding sites of the α subunit [14,16]. Many of these mutations have been found to disturb the function of the Na^+^,K^+^-ATPase by reducing its Na^+^ affinity [16,27,35,36,37], which in vivo will result in a critical lowering of the transport rate, as the intracellular concentration of Na^+^ is much lower than saturating under physiological conditions in cells, even for the wild type Na^+^,K^+^-ATPase. A prime example is the α3 mutation α3-D923N, which causes a more than 100-fold reduction in Na^+^ affinity [35]. The α3-D923N mutation is associated with RDP and AHC as well as intermediate RDP-AHC phenotypes [15,38,39,40]. More recently, the corresponding α1 mutation D933N has been shown to cause developmental delay accompanied by sleep disorder [25].

Here we describe for the first time super-efficient rescue mutations that fully compensate the reduced Na^+^ affinity in the human disease mutant α3-D923N to wild-type level. We likewise demonstrate a super-efficient rescue of Na^+^ affinity in α1-D928N, the rat equivalent of the human disease mutant α1-D933N (the reason for studying the rat version of α1-D933N is its low affinity for the inhibitor ouabain that provides an experimental advantage; see Results, Section 2.1, and Materials and Methods, Section 4.1). Our results elucidate the molecular mechanism underlying the rescue. Paradoxically, this insight evolved from our investigation of a rapid-onset ataxia (ROA) Na^+^,K^+^-ATPase mutant, α3-G316S [41], which exhibits an increased affinity for Na^+^. Taking into consideration the above-described reduced Na^+^ affinity of several other neurological disease mutants, it is surprising to find that the α3-G316S mutation increases Na^+^ affinity. However, when applied as second-site mutations, α3-G316S and the corresponding rat α1-G321S turned out to be super-efficient rescuers of the reduced Na^+^ affinity of Na^+^,K^+^-ATPase disease mutant α3-D923N and the corresponding rat α1-D928N. We furthermore demonstrate a similarly strong rescuing power of mutations breaking a salt bridge present in the K^+^-bound E_2_ state of the Na^+^,K^+^-ATPase but not in the Na^+^-bound E_1_ state (cf. the Na^+^,K^+^-ATPase reaction cycle in Figure 1B). Altogether, these findings provide compelling evidence for the molecular mechanism underlying the rescue effect. It is anticipated that this insight will be of value in the development of efficient medications to treat patients with Na^+^,K^+^-ATPase disease mutations that reduce the affinity for Na^+^.

## 2. Results

### 2.1. All Mutants Support Cell Growth

The present study encompasses a total of 24 mutations of the α1 and α3 isoforms of Na^+^,K^+^-ATPase, as well as the corresponding α1- and α3-wild types (Table 1). Mutations were introduced in cDNAs encoding ouabain-insensitive versions of these isoforms [42,43,44]. Hence, we used the rat α1 isoform in studies of α1-mutants and the α1-wild type. In the studies of α3-mutants and the α3-wild type, we used the human α3 isoform made ouabain-insensitive by mutations Q108R and N119D, inserting the same residues as present at these positions in rat α1 (see further in Materials and Methods, Section 4.1). In the following, residue numbering of α1-mutants and the α1-wild type is therefore according to rat α1, and residue numbering of α3-mutants and the α3-wild type is according to human α3 (5 lower than rat α1).

After transfection of COS-1 cells with the respective cDNAs, it was examined whether the mutants could support cell growth in the presence of ouabain at a concentration inhibiting the ouabain-sensitive endogenous COS-1 cell Na^+^,K^+^-ATPase. Under these conditions, the survival of the cells depends on the expression level and functional capability of the mutants, because the ouabain-sensitive endogenous COS-1 cell Na^+^,K^+^-ATPase is inactive [42,43,44]. In addition to the α1- and α3-wild types, all the 24 mutants examined were able to support cell growth in the presence of ouabain, indicating that a certain level of Na^+^ and K^+^ transport activity was retained with the mutation. According to our previous experience with a variety of Na^+^,K^+^-ATPase mutants, the transfected COS-1 cells are unable to survive under the applied ouabain selection pressure if the mutant exhibits less than 5% of the activity of a transfected wild type [16]. Stable cell lines were isolated and grown under the ouabain selection pressure. Plasma membranes were prepared from these cells and made permeable to allow access of substrates from both sides of the membrane as required for functional characterization of the mutant Na^+^,K^+^-ATPases. Based on the capacity for phosphorylation from ATP (“active site concentration”, see Materials and Methods), the expression levels of the mutants were in most cases similar to or even higher than that of the corresponding wild type (Table 1).

### 2.2. Mutations α3-G316S and α1-G321S Increase Na^+^ Affinity

**Figure 2:**
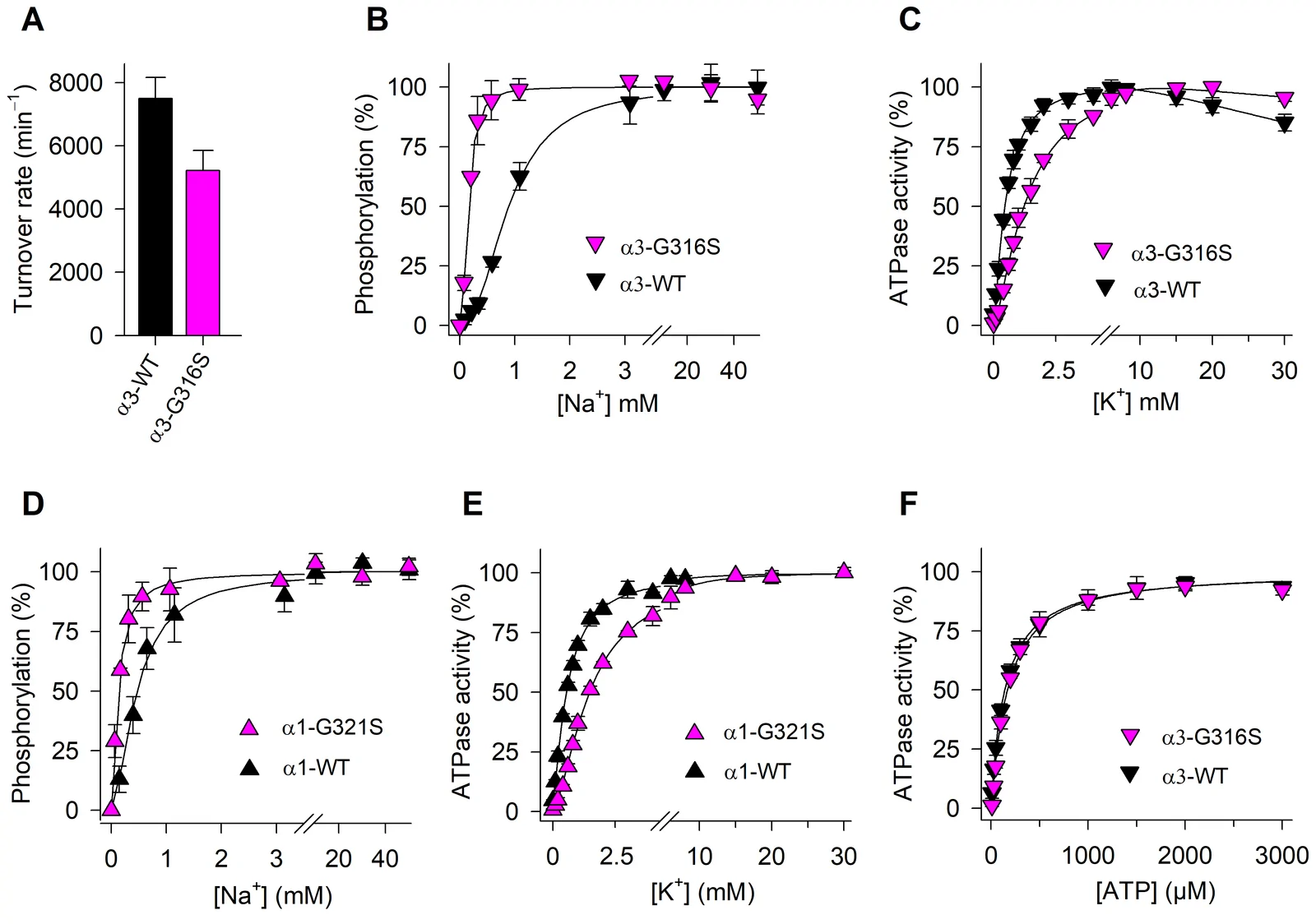
Functional changes caused by α3-G316S and α1-G321S mutations. (**A**) Catalytic turnover rate for ATPase activity (the number of enzyme cycles per molecule per min) of α3-G316S and α3-wild type at 130 mM Na^+^, 20 mM K^+^ and 3 mM ATP. (**B**) Na^+^ concentration dependence of phosphorylation from ATP of α3-G316S and α3-wild type in the absence of K^+^ and presence of oligomycin to slow dephosphorylation. (**C**) K^+^ concentration dependence of ATPase activity of α3-G316S and α3-wild type in the presence of 40 mM Na^+^ and 3 mM ATP. (**D**,**E**) Na^+^ and K^+^ dependences of α1-G321S and α1-wild type determined under the same conditions as applied for panels (**B**,**C**). **(F**) ATP concentration dependence of ATPase activity of α3-G316S and α3-wild type in the presence of 130 mM Na^+^ and 20 mM K^+^. For turnover rates of both α3 (panel (**A**)) and α1 variants and extracted K_0.5_Na^+^ values (panels (**B**,**D**)) with statistics, see Table 2. The extracted K_0.5_K^+^ and K_0.5_ATP values (panels (**C**,**E**,**F**)) are: K_0.5_K^+^ α3-wild type 0.438 ± 0.039 mM (*n* = 6), K_0.5_K^+^ α3-G316S 1.201 ± 0.098 mM (*n* = 3), K_0.5_K^+^ α1-wild type 0.589 ± 0.007 mM (*n* = 3), K_0.5_K^+^ α1-G321S 1.490 ± 0.020 mM (*n* = 3), K_0.5_ATP α3-wild type 0.144 ± 0.019 mM (*n* = 7), K_0.5_ATP α3-G316S 0.167 ± 0.007 mM (*n* = 3).

We set out by examining the disturbance of Na^+^,K^+^-ATPase function caused by the rapid-onset ataxia (ROA) mutation α3-G316S found in a patient presenting clinically with ataxia associated with cerebellar atrophy [41]. Moreover, the patient had symptoms overlapping to some extent with rapid-onset dystonia parkinsonism (RDP), but without dystonia, a defining symptom of RDP. The glycine residue α3-G316 is located rather close to Na^+^ and K^+^ binding site II, in the helical part of transmembrane segment M4 connecting site II with the extracellular border (M4E in Figure 1C,D). We also studied the corresponding rat α1 mutant, α1-G321S. Both mutants were able to form a phosphoenzyme intermediate in the presence of Na^+^ and ATP and displayed Na^+^,K^+^-ATPase activity. Based on the capacity for phosphorylation from ATP, the expression level of α3-G316S and α1-G321S in the COS-1 cells was higher than the respective α3- and α1-wild type levels (Table 1). The turnover rate of the Na^+^,K^+^-ATPase enzyme cycle (i.e., Na^+^,K^+^-ATPase ATP hydrolysis rate per active site, see Materials and Methods) determined under maximal conditions for the wild type (130 mM Na^+^, 20 mM K^+^, and 3 mM ATP) was 70% and 75% for α3-G316S and α1-G321S relative to the respective α3- and α1-wild types (Figure 2A and Table 2), i.e., only moderately reduced.

**Figure 3:**
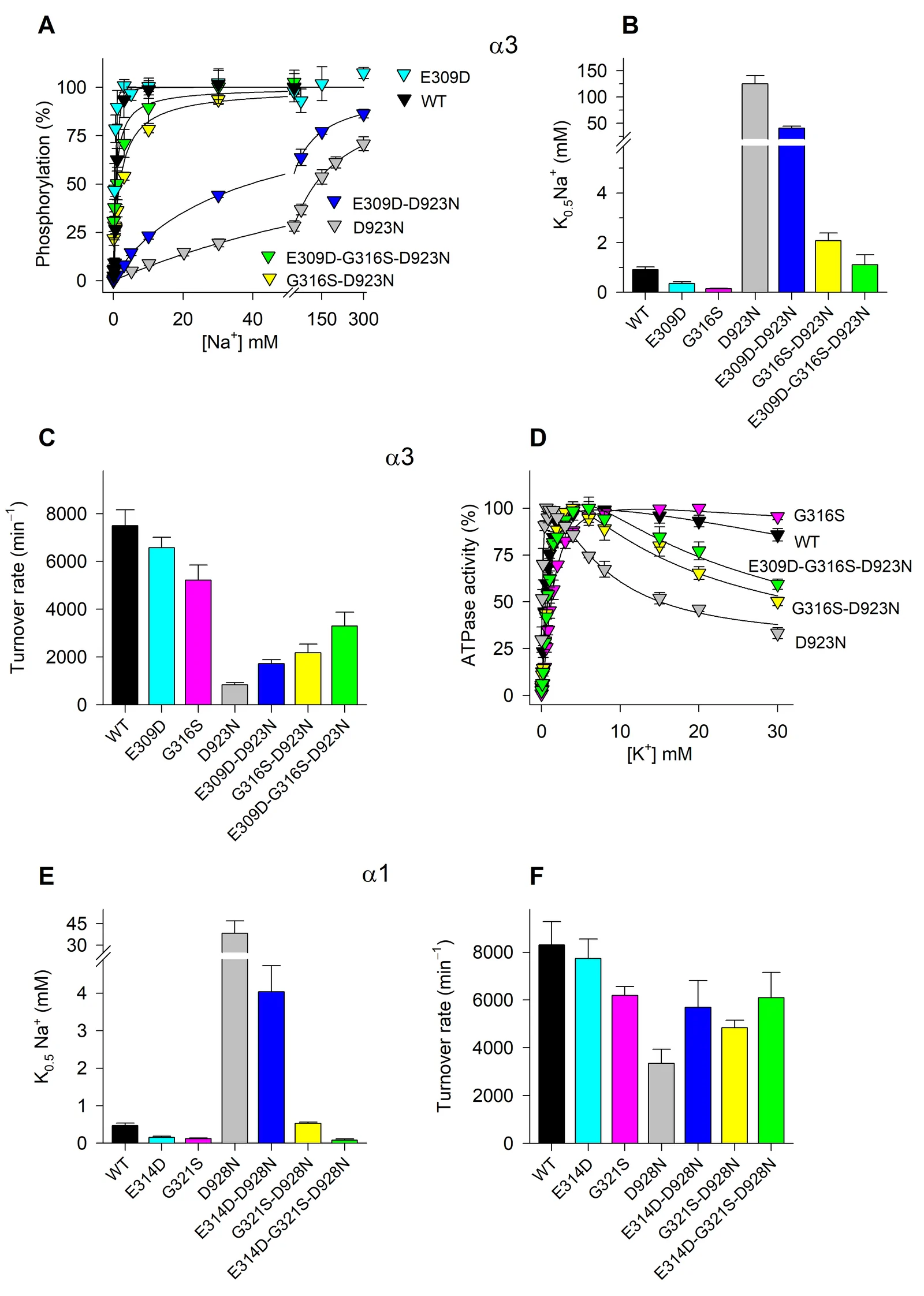
Rescue of Na^+^ affinity and catalytic turnover rate of α3-D923N and α1-D928N mutants by second-site mutations α3-G316S and α1-G321S in combination with α3-E309D and α1-E314D. (**A**) Na^+^ concentration dependence of phosphorylation from ATP of the indicated α3 variants under conditions as for Figure 2B. (**B**) K_0.5_Na^+^ values extracted from the data in panel (**A**) and Figure 2B. (**C**) Catalytic turnover rates for ATPase activity of the same α3 variants under conditions as for Figure 2A. (**D**) K^+^ concentration dependence of ATPase activity of the same α3 variants under conditions as for Figure 2C. (**E**) K_0.5_Na^+^ values of the indicated α1 variants determined under conditions as for panels (**A**,**B**). (**F**) Catalytic turnover rates for ATPase activity of the same α1 variants under conditions as for as for Figure 2A. For extracted K_0.5_Na^+^ values and turnover rates with statistics, see Table 2.

The phosphoryl transfer from ATP (reaction 3 in Figure 1B) requires Na^+^ binding at the three sites of the E_1_ state (reaction 2 in Figure 1B). Hence, we evaluated the Na^+^ affinity from the Na^+^ dependence of phosphorylation from ATP. The apparent affinity for Na^+^ is increased (K_0.5_Na^+^ reduced) 6.5-fold and 3.9-fold for α3-G316S and α1-G321S relative to the respective wild types (Figure 2B,D, Table 2). The activation of Na^+^- and K^+^-dependent ATPase activity by K^+^ shown in Figure 2C,E reflects the stimulation of dephosphorylation through K^+^ binding at the extracellular-facing sites of the K^+^-sensitive E_2_P intermediate (reactions 5 and 6 in Figure 1B). For α3-G316S and α1-G321S, the apparent K^+^ affinity for the stimulation is 2.7-fold and 2.5-fold reduced (K_0.5_K^+^ increased) relative to the respective wild types. K^+^ at the highest concentrations furthermore causes inhibition of the Na^+^,K^+^-ATPase activity in the α3-wild type (Figure 2C). This inhibition may be explained by the binding of K^+^ at the cytoplasmic-facing E_1_ sites in competition with Na^+^ [1,27], which leads to conversion of E_1_ to the K^+^-occluded E_2_(K_2_) form, by reversal of reaction 1 in Figure 1B. Because the affinity for Na^+^ has increased in α3-G316S relative to the α3-wild type, the inhibition by K^+^ is much less pronounced in the mutant. Likewise, no K^+^ inhibition is seen for the α1-wild type and α1-G321S, which both exhibit higher Na^+^ affinity than the α3-wild type (see Figure 3D for examples of a very pronounced inhibition by K^+^ of Na^+^,K^+^-ATPase activity in mutants with a strongly reduced Na^+^ affinity). Of note is moreover that the increased Na^+^ affinity and reduced affinity for K^+^ stimulation of the activity resulting from the glycine mutation seems not to be associated with any change of the apparent affinity for ATP, as the ATP concentration dependence of Na^+^,K^+^-ATPase activity is almost unaffected by the α3-G316S mutation (Figure 2F).

### 2.3. α3-G316S and α1-G321S Are Super-Efficient Rescue Mutations

Unlike the above-described ROA mutation, several other neurological disease mutations reduce the affinity of the Na^+^,K^+^-ATPase for Na^+^ [16,27,35,36,37]. A dramatic, more than 100-fold reduction in Na^+^ affinity has been reported for the α3-D923N mutation [35], which is associated with the neurological disorders RDP and AHC [15,38,39,40]. Likewise, ~80-fold reduction in Na^+^ affinity has been observed for the rat α1-D928N mutation [36], which is the rat equivalent of the human α1-D933N mutation associated with developmental delay accompanied by sleep disorder [25]. These mutations target an aspartate in Na^+^ site III (see Figure 1C). The reduced Na^+^ affinity of the α1-D928N mutant was found to be partially rescued (~10-fold improvement of Na^+^ affinity) by introduction of a second-site mutation, α1-E314D, located in M4E, far away in the structure from Na^+^ site III [36] (E309D by the α3 numbering of residues shown in Figure 1). This finding suggested that further exploration of the recue principle with elucidation of the underlying molecular mechanism might help create a platform for a rational therapeutic approach to restore the transport activity of Na^+^,K^+^-ATPase with defective Na^+^ binding.

In Figure 3A,B and Table 2 we present Na^+^ concentration dependences of phosphorylation and corresponding K_0.5_Na^+^ values for combinations of the disease mutation α3-D923N with second-site mutations α3-E309D (α3 mutation corresponding to α1-E314D) and α3-G316S, taking advantage of the increased Na^+^ affinity described above for α3-G316S. By itself, the disease mutation α3-D923N reduces Na^+^ affinity by as much as 135-fold ([35] and Table 2). Introduction of α3-E309D as second-site mutation increases Na^+^ affinity only moderately (~3-fold), thus leaving a considerable >40-fold difference in Na^+^ affinity from the α3-wild type. Remarkably, α3-G316S has a much more dramatic rescuing effect than α3-E309D, increasing the Na^+^ affinity of α3-D923N as much as ~60-fold (K_0.5_Na^+^ reduced from ~120 mM to 2 mM), such that the Na^+^ affinity of α3-G316S-D923N is only ~2-fold lower than that of the α3-wild type. Moreover, the triple mutant α3-E309D-G316S-D923N, combining α3-D923N with α3-G316S as well as α3-E309D, completely restores Na^+^ affinity to a wild-type-like level (Figure 3A,B and Table 2). The glutamate E309 is positioned two helix-turns below G316 on the same side of the M4E helix but further away from Na^+^-binding site II, closer to the extracellular border (Figure 1A,C,D). Figure 3C shows that the turnover rate of the Na^+^,K^+^-ATPase enzyme cycle increases in the same order as the apparent Na^+^ affinities: α3-D923N (11% of the α3-wild type) < α3-E309D-D923N (23% of the α3-wild type) < α3-G316S-D923N (29% of the α3-wild type) < α3-E309D-G316S-D923N (44% of the α3-wild type). The latter triple mutant exhibits a turnover rate ~4-fold higher than that of α3-D923N.

Figure 3D depicts the K^+^ concentration dependence of Na^+^,K^+^-ATPase activity. For α3-D923N and composite mutants containing α3-D923N, two distinct phases are present. As explained above in relation to Figure 2C, the activation phase is due to stimulation of dephosphorylation (reactions 5 and 6 in Figure 1B) and, thus, stimulation of the whole Na^+^,K^+^-ATPase cycle, by K^+^ binding to the E_2_P intermediate, whereas the inhibition reflects binding of K^+^ to E_1_ in competition with Na^+^, thus reversing reaction 1 in Figure 1B. The inhibition is most pronounced for α3-D923N and decreases in the order α3-D923N > α3-G316S-D923N > α3-E309D-G316S-D923N > α3-wild type, thus reflecting the gradual increase in Na^+^ affinity.

The Na^+^ affinities of the α1 mutants corresponding to the α3 mutants described above follow the same pattern as for the α3 mutants. Hence, α1-G321S increases the low Na^+^ affinity of α1-D928N ~70-fold, nearly reaching the α1-wild type level. The combined triple mutant, α1-E314D-G321S-D928N, possesses even higher Na^+^ affinity (6-fold) than the α1-wild type (Figure 3E and Table 2). The turnover rate of the Na^+^,K^+^-ATPase enzyme cycle also increases with rescue by α1-E314D (from 40% of the α1-wild type to 69%), but the α1-G321S mutation does not add significantly to this increase, as the turnover rate of the α1-E314D-G321S-D928N triple mutant is 73% that of the α1-wild type (Figure 3F and Table 2).

### 2.4. Super-Efficient Rescue Can Also Be Obtained by Replacing α3-E309 or α1-E314 with Neutral and Positively Charged Residues

**Figure 4:**
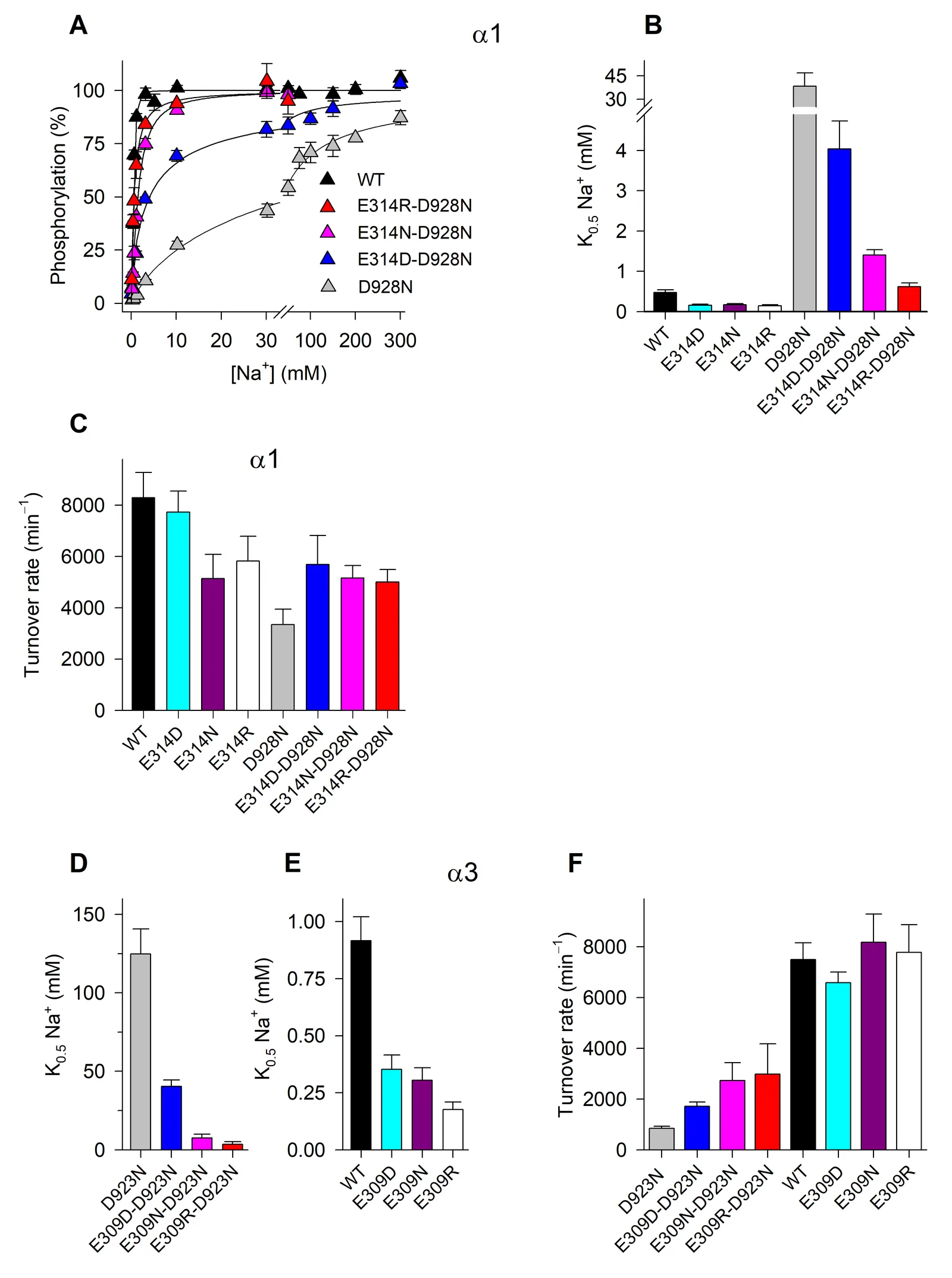
Rescue of Na^+^ affinity and catalytic turnover rate of α1-D928N and α3-D923N mutants by second-site mutations of α1-E314 or α3-E309 that affect salt bridge formation with α1-R882 and α3-R877, respectively. (**A**) Na^+^ concentration dependence of phosphorylation from ATP of the indicated α1 variants under conditions as for Figure 2B. (**B**) K_0.5_Na^+^ values extracted from the data in panel (**A**) and from data obtained under similar conditions for the mutants E314D, E314N and E314R with single substitution. (**C**) Catalytic turnover rates for ATPase activity of the same α1 variants under conditions as for Figure 2A. (**D**,**E**) K_0.5_Na^+^ values of the indicated α3 variants determined under conditions as for panels (**A**,**B**). Note the different ordinate scales in panels (**D**,**E**). (**F**) Catalytic turnover rates for ATPase activity of the same α3 variants under conditions as for panel (**C**). For extracted K_0.5_Na^+^ values and turnover rates with statistics, see Table 2.

In the structure of the K^+^-bound E_2_ form of the wild type (Figure 1D), the glutamate α3-E309/α1-E314 of M4E appears to form a salt bridge with an arginine, α3-R877/α1-R882, in the extracellular loop between transmembrane segments 7 and 8 (“L7-8” loop). Because replacement of the glutamate with aspartate reduces the length of the side chain by one carbon atom, i.e., by ~1 Å, this mutation is likely to weaken or break the salt bridge, which might form the basis for the partial rescue of Na^+^ affinity by mutation α3-E309D/α1-E314D. This consideration prompted us to further examine the effects of disrupting the salt-bridge by replacing α1-E314 and α3-E309 with a neutral asparagine or positively charged arginine in the α1-D928N and α3-D923N mutants as well as in the α1- and α3-wild types (Figure 4). The rescue of Na^+^ affinity is clearly much more efficient with α1-E314N or α1-E314R as a second-site mutation than with α1-E314D (Figure 4A,B, and Table 2). Relative to α1-D928N, Na^+^ affinity is improved ~10-fold in α1-E314D-D928N, ~30-fold in α1-E314N-D928N, and ~60-fold in α1-E314R-D928N (Na^+^ affinity reduced ~80-fold, 9-fold, 3-fold-, and 1.3-fold for α1-D928N, α1-E314D-D928N, α1-E314N-D928N, and α1-E314R-D928N, respectively, relative to the α1-wild type). Hence, Na^+^ affinity increases in the following order: α1-D928N < α1-E314D-D928N < α1-E314N-D928N < α1-E314R-D928N ~ α1-WT. Thus, with α1-E314R as a second-site mutation, i.e., the negatively charged glutamate side chain being replaced by a positively charged arginine, Na^+^ affinity is rescued almost to wild-type level. Na^+^ affinity is also increased relative to the α1-wild type in the mutants carrying a rescue mutation as single substitution, α1-E314D, α1-E314N, and α1-E314R. However, in these cases the increase is more modest compared with the effects on Na^+^ affinity of α1-D928N and almost equal (3-fold) for the three singly substituted mutants (Figure 4B and Table 2). The turnover rate of the Na^+^,K^+^-ATPase enzyme cycle of α1-D928N is 40% that of the α1-wild type. Introduction of α1-E314D, α1-E314N, or α1-E314R as a second-site mutation in α1-D928N rescues the turnover rate of α1-D928N to levels ranging between 60% and 69% that of the α1-wild type, which is similar to the turnover rate observed for the single mutations α1-E314N and α1-E314R (Figure 4C and Table 2). Hence, the reduced turnover rate of α1-E314N and α1-E314R relative to the α1-wild type seems to be the limiting factor for the increase in turnover rate that can be achieved by the rescue.

In α3, Na^+^ affinity is improved ~3-fold, 17-fold, and ~34-fold in α3-E309D-D923N, α3-E309N-D923N, and α3-E309R-D923N, respectively, relative to α3-D923N (Figure 4D and Table 2). The Na^+^ affinity of α3-E309R-D923N is only ~4-fold reduced relative to the α3-wild type. The mutants with a rescue mutation as a single substitution, α3-E309D, α3-E309N, and α3-E309R, also show an increase in Na^+^ affinity, from ~3-fold to ~5-fold relative to the α3-wild type, with the Na^+^ affinity increasing in the same order as in the double mutants: α3-E309D < α3-E309N < α3-E309R (Figure 4E and Table 2). There is an increasing rescue effect of the second-site mutations on the turnover rate of the Na^+^,K^+^-ATPase enzyme cycle of α3-D923N (Figure 4F and Table 2), again in the order α3-D923N (11% of the α3-wild type) < α3-E309D-D923N (23% of the α3-wild type) < α3-E309N-D923N (36% of the α3-wild type) < α3-E309R-D923N (40% of the α3-wild type). The α3 mutants with a single substitution, α3-E309D, α3-E309N, and α3-E309R, all exhibit a turnover rate indistinguishable from that of the α3-wild type (Figure 4F and Table 2).

### 2.5. Rescue Effect of Disruption of the Salt Bridge by Alanine Substitution of α1-R882 or α3-R877

**Figure 5:**
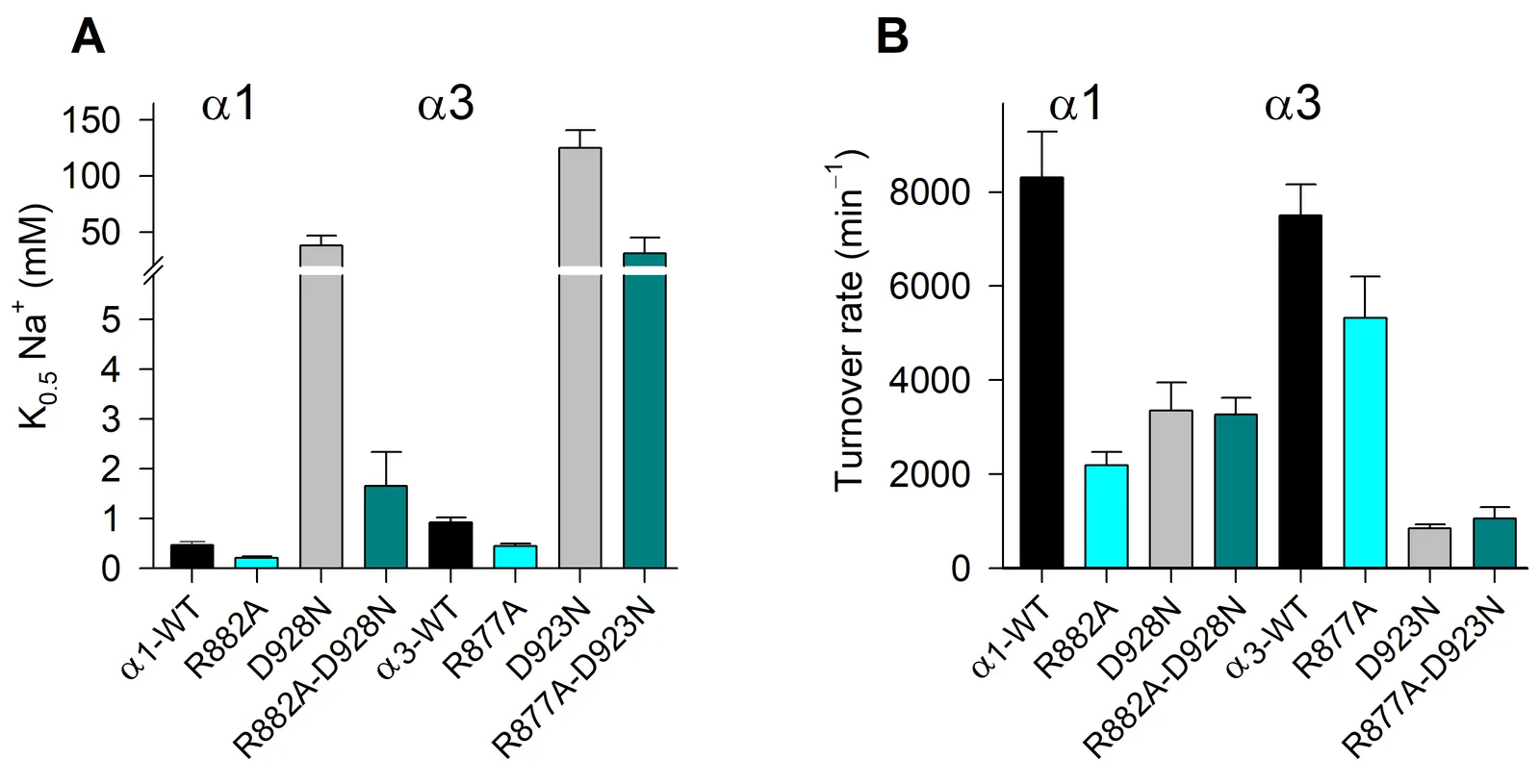
Rescue of Na^+^ affinity and effect on catalytic turnover rate of α1-D928N and α3-D923N mutants by second-site mutations α1-R882A and α3-R877A. (**A**) K_0.5_Na^+^ values of the indicated α1 and α3 variants determined under conditions as for Figure 2B. (**B**) Catalytic turnover rates for ATPase activity of the same α1 and α3 variants under conditions as for Figure 2A. For extracted K_0.5_Na^+^ values and turnover rates with statistics, see Table 2.

To further examine the hypothesis that disruption of the salt bridge between the M4E glutamate and the arginine in the L7-8 extracellular loop constitutes an essential part of the molecular mechanism underlying the rescue effects, we replaced the arginine in the salt bridge by alanine in the α1-D928N and α3-D923N mutants as well as in the α1- and α3-wild types (Figure 5).

With α1-R882A as a second-site mutation in the α1-D928N mutant, Na^+^ affinity is improved 23-fold, being only 3.5-fold lower than that of the wild type. With α3-R877A as a second-site mutation in the α3-D923N mutant, Na^+^ affinity is improved 4-fold, being 34-fold lower than that of the wild type (Figure 5A and Table 2). Hence, the rescuing effect on Na^+^ affinity of α1-R882A (23-fold improvement) is considerably stronger than that of α1-E314D (10-fold improvement), and the rescuing effect of α3-R877A (4-fold improvement) is slightly better than that of α3-E309D (3-fold improvement). On the other hand, in α1-D928N as well as α3-D923N, the turnover rate of the Na^+^,K^+^-ATPase enzyme cycle is unaffected by the alanine substitution of the arginine (39% of the α1-wild type versus 40% of the α1-wild type and 14% of the α3-wild type versus 11% of the α3-wild type, respectively). In the mutants with single substitution of the arginine alone, α1-R882A and α3-R877A, the turnover rate is considerably reduced, to 26% and 71%, respectively, of the corresponding wild type (Figure 5B and Table 2), thus indicating that the arginine plays an important role in another part of the Na^+^,K^+^-ATPase enzyme cycle as well (see further in Discussion).

## 3. Discussion

### 3.1. The Rapid-Onset Ataxia (ROA) Na^+^,K^+^-ATPase Mutant α3-G316S and the Corresponding α1-G321S Display Increased Na^+^ Affinity

In contrast to several neurological disease mutations known to reduce the affinity of Na^+^,K^+^-ATPase for Na^+^, as exemplified by α3-D923N, we here report that the α3-G316S mutation and the corresponding α1-G321S cause considerable increases in Na^+^ affinity, 6.5-fold and 3.9-fold, respectively. The α3-G316S mutation was found as a de novo variant in a patient with rapid-onset ataxia (ROA). How the increased Na^+^ affinity relates to the neurological phenotype remains an open question, but in addition to α3-G316S, a second de novo variant, in the ubiquitin pathway component ubiquilin 4 (UBQLN4), was also present in this patient and may have contributed to the progressive cerebellar atrophy causing ROA [41]. Of note is also that the catalytic turnover rate of the α3-G316S mutant is only moderately reduced to 70% of the wild-type level and that its expression level in our COS-1 cell expression system is higher than that of the wild type. In fact, the absence of dystonia, the cardinal symptom of rapid-onset dystonia parkinsonism (RDP), from this patient’s spectrum of symptoms might be related to the lack of the detrimental effects on the ability to transport Na^+^ seen for several RDP mutations. The increased Na^+^ affinity of α3-G316S is, in any case, of great interest in the context of trying to understand how the reduced Na^+^ affinity found in several other Na^+^,K^+^-ATPase neurological disease mutants can be rescued. Hence, we opted to examine whether α3-G316S and the corresponding rat α1-G321S could be used as second-site mutations to rescue the reduced Na^+^ affinity caused by the neurological disease mutation α3-D923N and the corresponding rat α1-D928N.

### 3.2. Super-Efficient Rescue of Na^+^ Affinity in Disease Mutant α3-D923N and α1-D928N by Specific Second-Site Mutations

The Na^+^,K^+^-ATPase α3-D923N mutation causes rapid-onset dystonia parkinsonism (RDP) and alternating hemiplegia of childhood (AHC) as well as intermediate RDP-AHC phenotypes [15,38,39,40]. Recently, the corresponding Na^+^,K^+^-ATPase α1 mutation, α1-D933N (human equivalent of the rat α1-D928N examined in the present study), was found associated with developmental delay accompanied by sleep disorder [25]. The reduction in Na^+^ affinity resulting from these and several other neurological disease mutations [16,27,35,36,37] may result in a critical lowering of Na^+^,K^+^-pump activity in neurons, as the intracellular concentration of Na^+^ is far from saturating even for the wild type Na^+^,K^+^-pump, which is running only at a fraction of its maximum speed under physiological conditions in cells.

The aspartates replaced by asparagine in α3-D923N and α1-D928N are located at corresponding positions in the two isoforms, in transmembrane segment M8, where the aspartate is known to contribute to Na^+^ binding at the so-called Na^+^ site III [5,35,45]. In both isoforms, the mutation strongly reduces Na^+^ affinity, although the effect differs quantitatively: 135-fold reduction in Na^+^ affinity in α3-D923N, relative to the α3-wild type, and ~80-fold reduction in Na^+^ affinity in α1-D928N, relative to the α1-wild type (Table 2). Note also that the apparent Na^+^ affinity of the α3-wild type is ~2-fold lower than that of the α1-wild type, which is not immediately predictable, as the amino-acid residues known to contribute to Na^+^ binding are the same in α1 and α3. The difference in Na^+^ affinity between the α1- and α3-wild types may reflect a poise of the E_1_-E_2_ conformational equilibrium more toward E_2_ in α3 relative to α1, which is due to differences in other residues than those interacting directly with the ions [7].

Here, we demonstrate that the huge reductions in Na^+^ affinity caused by the α3-D923N and α1-D928N mutations can be rescued by α3-G316/α1-G321 and α3-E309/α1-E314 second-site mutations, both residues being located in M4E, the helical part of transmembrane segment M4 closest to the extracellular side, far away in the structure from Na^+^ site III (cf. Figure 1). Mutations α3-G316S/α1-G321S are super-efficient rescuers (Figure 3), as are mutations of α3-E309/α1-E314 that replace the negatively charged glutamate with a neutral asparagine or (even better) with the positively charged arginine (Figure 4). In the wild type, these mutations also increase Na^+^ affinity, but not to an extent comparable to what is seen when they are inserted as second-site mutations together with the neurological disease mutation in α3-D923N or α1-D928N. Shortening of the side chain of α3-E309 or α1-E314 by replacement with aspartate also has some rescuing effect, as previously reported for α1-E314D [36], although weaker than seen when removing the negative charge. However, when applied together, the rescuing effects of α3-G316S/α1-G321S and α3-E309D/α1-E314D lead to a wild type-like or even higher Na^+^ affinity (Figure 3).

### 3.3. Disruption of a Salt Bridge Rescues Na^+^ Affinity

A clue to understanding the structural basis for the rescue is obtained by considering the high-resolution atomic structures of the Na^+^-bound E_1_ form and the K^+^-bound E_2_ form (Figure 1) [5,6]. Notably, in the E_2_ form the glutamate α3-E309/α1-E314 of the M4E helix is engaged in a salt bridge with the arginine α3-R877/α1-R882 in the extracellular L7-8 loop and in hydrogen bonding with the threonine α3-T306/α1-T311 in M4E, whereas these bonds are broken in the E_1_ form, where the ion-binding pocket has acquired high affinity for Na^+^ due to displacement of the M4E helix toward the cytoplasmic side. Mutations that disrupt these bonds should therefore destabilize the E_2_ position of the M4E helix relative to the E_1_ position, thus leading to a higher Na^+^ affinity. Our results demonstrate that interference with the above-mentioned bonds indeed leads to an increase in Na^+^ affinity. The α3-E309D/α1-E314D mutation will at least to some extent affect the salt bridge to the arginine, due to shortening of the side chain of the glutamate by one carbon atom (corresponding to ~1 Å) leading to movement of M4E toward the cytoplasmic side, i.e., in the same direction as when E_2_ is turned into E_1_ during the normal Na^+^,K^+^-ATPase transport cycle [45]. However, the insertion of the neutral asparagine should be a better salt-bridge breaker, and the insertion of the arginine in place of the glutamate should have an even larger effect, because the two positively charged arginine side chains at positions α3-R309/α1-R314 and α3-R877/α1-R882 undergo electrostatic repulsion (and possibly also clash sterically due to the large size of the arginine side chain). Hence, it is understandable that the rescue increases in efficiency from aspartate through asparagine to arginine. That disruption of the salt bridge constitutes an important part of the rescue mechanism is underscored by the partial rescue of Na^+^ affinity obtained also by alanine substitution of α3-R877/α1-R882 (Figure 5A). The rescue effect on Na^+^ affinity is in this case of a magnitude between those of α3-E309D/α1-E314D and α3-E309N/α1-E314N, predictably smaller than for the α3-E309R/α1-E314R mutation, because there is no electrostatic repulsion or steric clash and no interference with the interaction of α3-E309/α1-E314 with the threonine α3-T306/α1-T311.

### 3.4. Na^+^ Affinity Is Also Rescued by Creating a Clash Between Membrane Helices

**Figure 6:**
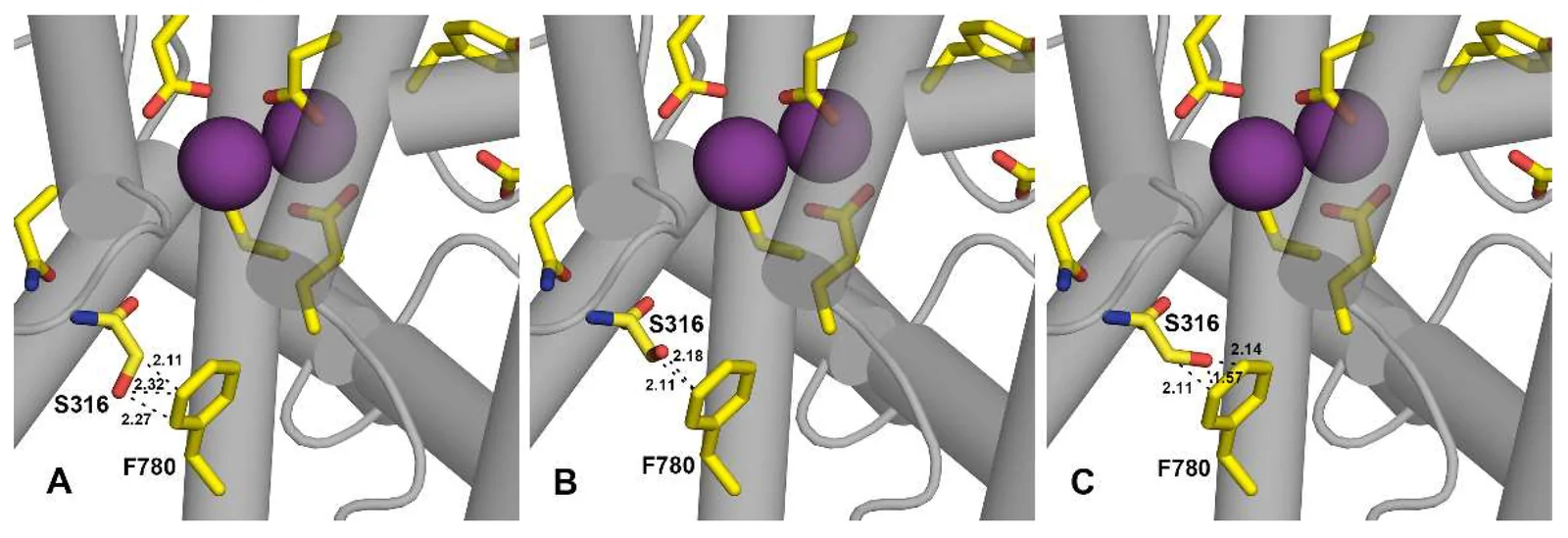
Structural modeling of the K^+^-bound E_2_ state showing clash between the serine inserted in place of the M4 glycine α3-G316/α1-G321 and the M5 phenylalanine α3-F780/α1-F785 (both are in the panels numbered according to α3, i.e., S316 and F780). Panels (**A**–**C**) correspond to the three possible rotamers of serine, which have been inserted in the structure with PDB ID 2ZXE also used in Figure 1D (E_2_∙P_i_(K_2_) form, K^+^ shown as purple spheres, carbon atoms yellow, nitrogen atoms blue, oxygen atoms red). As indicated by the dotted lines with numbers showing distances in Å, several distances from the serine side-chain carbon or oxygen to the phenyl ring carbons are in the range 1.6–2.2 Å. Given van der Waals radii as typically found in proteins of 1.7 Å for carbon in serine side chains or phenyl rings and 1.5 Å for serine side-chain oxygen, the contact distances are about 1.7 Å + 1.7 Å = 3.4 Å for clash of the serine side chain β-carbon with phenyl ring carbon and for clash of the serine side-chain oxygen with phenyl ring carbon 1.5 Å + 1.7 Å = 3.2 Å. The distances calculated for the mutant assuming a normal E_2_ conformation as shown in the panels are, thus, shorter than the contact distances by at least 1 Å, thus predicting a clash with resulting destabilization of the E_2_ state. The corresponding distances in the Na^+^-bound E_1_ state are several Å longer than the calculated contact distances, leading to the conclusion that introduction of the serine destabilizes the E_2_ state relative to the E_1_ state.

Then, how can the substitution of α3-G316/α1-G321 in the M4E helix with serine lead to a similar or even stronger rescue effect? Again, understanding is helped by comparing the E_1_ and E_2_ structures. In the E_2_ state, α3-G316/α1-G321 is rather close to a bulky phenylalanine α3-F780/α1-F785 in transmembrane segment M5. Substitution of the glycine with the larger serine will lead to a clash with the phenylalanine as outlined in Figure 6 and was previously predicted [41]. Because the clash will not occur in E_1_, where M4E is shifted toward the cytoplasmic side, the mutation will destabilize the E_2_ position of M4E relative to the E_1_ position, leading to higher Na^+^ affinity, just like the breakage of the salt bridge discussed above. Hence, the replacement of the glycine and the breakage of the salt bridge cooperate.

It should be noted that an E_1_ shift occurs not only when rescuing the effect of the neurological disease mutation α3-D923N/α1-D928N but also to some extent upon the single substitution of α3-G316/α1-G321 or α3-E309/α1-E314 in the wild type, as evidenced by the increased Na^+^ affinity relative to wild type observed in these cases. Importantly, the E_1_ shift appears to be restricted to the membrane sector harboring the ion binding sites and does not involve the ATP binding site in the cytoplasmic sector of the protein, because the apparent affinity for ATP stimulation of Na^+^,K^+^-ATPase activity is almost unaffected by the α3-G316S (Figure 2F) and α1-E314D [36] mutations. ATP binds with high affinity to E_1_, phosphorylating the enzyme in the presence of Na^+^ (reaction 3 in Figure 1B), and binds with low affinity to E_2_(K_2_) in a non-phosphorylating allosteric mode, accelerating the E_2_(K_2_) to E_1_ transition (reaction 1 in Figure 1B, red ATP) [2,3]. Hence, a global conformational change toward E_1_, involving the ATP binding site is expected to increase the apparent affinity for ATP stimulation of the Na^+^,K^+^-ATPase enzyme cycle, which was not observed.

In accordance with our interpretation of the effects of the α3-G316S/α1-G321S mutations in terms of a clash between the serine replacing the M4 glycine and the M5 phenylalanine, the opposite effect, a reduction in Na^+^ affinity, has been observed for mutants α1-F785L and α3-F780L, where the phenylalanine is replaced by a less bulky/more flexible residue, thus reducing the chance of a clash between the transmembrane segments M4E and M5 [27,37]. Also in this case was the apparent affinity for ATP stimulation of the Na^+^,K^+^-ATPase enzyme cycle unaffected, thus indicating a local change to the ion binding region in the membrane rather than a global conformational shift from E_1_ toward E_2_ involving the cytoplasmic sector [37].

### 3.5. Structural Interpretation in Relation to Cooperative Na^+^ Binding

It appears that the bonding network in E_2_ close to the extracellular side, involving α3-E309/α1-E314, anchors the M4E helix, and when this network is broken, and/or the M5 phenylalanine α3-F780/α1-F785 clashes with the serine replacing α3-G316/α1-G321, the M4E helix will move toward the cytoplasmic side with consequences for the ion binding pocket as in the normal Na^+^,K^+^-ATPase transport cycle, where such movement is part of the E_2_-to-E_1_ conformational transition [45]. Probably, what is important here is Na^+^ site II, which is located almost "on" the cytoplasmic end of M4E, with the glutamate α3-E324/α1-E329 as well as M4E backbone carbonyl groups contributing oxygen ligands for Na^+^ binding [5]. Hence, the movement of M4E induced by the rescue mutations may work by tightening the interaction with Na^+^ at site II. In fact, the α3-G316/α1-G321 glycine residue is located only about 9 Å from the Na^+^ ion at site II, thus allowing the clash of the serine substituent with the M5 phenylalanine α3-F780/α1-F785 to affect Na^+^ site II profoundly. Such an effect on site II will explain that Na^+^ activation of phosphorylation is greatly improved. Normally, the cooperativity in Na^+^ binding ensures that the Na^+^ affinity of site II increases when site III becomes occupied by Na^+^ [5,7]. This is not the case if the cooperativity is lost due to mutation of site III. However, when the Na^+^ affinity of site II instead is increased by the movement of M4E caused by the rescue mutations, the apparent Na^+^ affinity for activation of phosphorylation is improved, because a proper, tight binding of Na^+^ at site II is crucial for phosphorylation. Hence, the increase in the Na^+^ affinity of site II caused by the rescue mutations seems to bypass the need for the perfect interactions of site III residues with Na^+^ that normally make site II ready to bind Na^+^, thereby bringing the phosphorylation site aspartate in the P-domain in position to receive the γ-phosphate of ATP. Such a bypass would be in perfect agreement with a recent study implying that a mutation inserting a large and bulky residue that completely prevents binding of Na^+^ to site III still will allow Na^+^ to bind at site II and trigger the phosphorylation from ATP, because the mutation locks the membrane sector into an E_1_-like configuration [46].

### 3.6. Rescue of Turnover Rate

The rescue mutations also increase the turnover rate of the Na^+^,K^+^-ATPase enzyme cycle considerably in most cases. For α3-D923N, there is a distinct correlation between the rescue of Na^+^ affinity and the turnover rate (Figure 3C and Figure 4F). The turnover rate reflects the maximum ATPase activity per molecule in the wild type, where the Na^+^ concentration in the assay medium is saturating. However, in mutants with a substantial reduction in Na^+^ affinity, the Na^+^ concentration in the assay of ATPase activity (130 mM) is not saturating, because K^+^ (20 mM) is present as well (needed to bind to the two K^+^ sites in their extracellular-facing high-affinity configuration in E_2_P to activate the dephosphorylation that releases the phosphate, i.e., reactions 5 and 6 in Figure 1B). In the leaky membrane preparation used in our measurements, K^+^ competes with Na^+^ for binding to E_1_ sites I and II, but without triggering the forward phosphorylation from ATP as Na^+^ binding does. In the mutants with reduced Na^+^ affinity, this competition is sufficiently strong to lead to “backdoor” formation of the K^+^-occluded E_2_(K_2_) state (reaction 1 in Figure 1B, backwards), resulting in a reduced turnover rate. However, this effect will be counteracted by the destabilization of the K^+^-occluded E_2_(K_2_) state and by the promotion of Na^+^ binding at site II caused by the rescue mutation. Moreover, the rate of phosphorylation of the Na^+^-bound E_1_ state (reaction 3 in Figure 1B) may be reduced in mutants with defective Na^+^ binding, as observed previously for mutant α1-D928N [36]. In this case, the displacement of M4E toward the cytoplasmic side, optimizing the configuration of site II for Na^+^ binding [45], may be instrumental in the increase in the turnover rate by the rescue mutation through an increase in the phosphorylation rate. However, in some of the mutants the rescue of the turnover rate seems to be limited by a negative effect on the turnover rate caused by the rescue mutation itself. This is most clearly seen for α1-R882A and α3-R877A (Figure 5B), but also the less drastic reductions in turnover rates caused by the α1-G321S, α1-E314N, and α1-E314R mutations by themselves seem to pose limitations to how much rescue of the turnover rate of α1-D928N can be achieved with these as second-site mutations (Figure 3F and Figure 4C). In these cases, the reason for the negative effect on the turnover rate of the rescue mutation may be that the residue replaced by the rescue mutation plays an important role in another part of the Na^+^,K^+^-ATPase enzyme cycle. Hence, the arginine α1-R882/α3-R877 seems to form a salt bridge with an asparagine in the L5-6 extracellular loop in the E_2_P state [47], which will be disturbed by the alanine substitution introduced to break the salt bridge with the M4E glutamate in the E_2_(K_2_) state, with possible consequences for K^+^ binding and activation of dephosphorylation of E_2_P.

## 4. Materials and Methods

### 4.1. Site-Directed Mutagenesis and Expression

Base substitutions were introduced by PCR into the full-length cDNA encoding either the ouabain-insensitive rat α1 isoform of Na^+^,K^+^-ATPase or the human α3 isoform made ouabain-insensitive by mutations Q108R and N119D, inserting the same residues as present at these positions in rat α1 [35,42]. The intended mutations were verified by full-length sequencing before transfection into COS-1 cells, a monkey kidney fibroblast-like cell line acquired from the American Type Culture Collection (ATCC, CRL-1650; Manassas, VA, USA) [48,49]. The cells were grown in DMEM medium (VWR, HyClone Laboratories, South Logan, UT, USA) containing penicillin and streptomycin (50 units/mL each) (Life Technologies, Bleiswijk, The Netherlands), 1% non-essential amino acids (Life Technologies, Paisley, Scotland, UK), and 10% fetal bovine serum (HyClone Laboratories, South Logan, UT, USA), at 37 °C and 5% CO_2_. The Ca^2+^-phosphate precipitation method [50] was used for transfection. No exogenous β subunit was introduced, because the exogenous α1 subunit associates with the endogenous COS-1 cell β subunit. To enhance DNA uptake, the transfected cells were treated with 10% glycerol (Sigma-Aldrich, Saint Louis, MO, USA). The ouabain-selection methodology [42,43,44] was applied to obtain stable expression of the exogenous Na^+^,K^+^-ATPase with and without the introduced mutations. This expression strategy takes advantage of the relative insensitivity of the exogenous transfected Na^+^,K^+^-ATPase to the inhibitor ouabain as compared with the endogenous ouabain-sensitive COS-1 cell Na^+^,K^+^-ATPase. To initiate the selection of cells expressing the exogenous ouabain-insensitive Na^+^,K^+^-ATPase, 1 μM ouabain (Sigma-Aldrich, Darmstadt, Germany) was added to the DMEM medium three days after the transfection. The ouabain concentration was later increased to 5 μM. Because the latter concentration of ouabain inhibits the endogenous, ouabain-sensitive COS-1 cell Na^+^,K^+^-ATPase very efficiently, cells that do not express an exogenous active Na^+^,K^+^-ATPase die. Hence, survival of the transfected cells depends on the activity as well as the expression level of the exogenous ouabain-insensitive Na^+^,K^+^-ATPase. Like the α1- and α3-wild types, all the 24 mutants examined were able to support cell growth, thus conferring ouabain resistance to the cells, meaning that a certain level of function (see further in the Results Section 2.1) was retained by the mutants. The cells were grown under ouabain selection pressure until harvesting and isolation of plasma membranes containing the expressed enzyme. The intended mutations were verified again by repeated genomic sequencing throughout the growth period, during which the exogenous Na^+^,K^+^-ATPase DNA becomes stably integrated into the genome of the ouabain-resistant COS-1 cells. The exogenous DNA was amplified by PCR, using primers that span exon-exon boundaries, thereby annealing specifically to the exogenous DNA and not to the endogenous Na^+^,K^+^-ATPase DNA, which contains introns.

### 4.2. Preparation of Plasma Membrane Vesicles

Crude plasma membrane vesicles from the cells containing the expressed wild type or mutant enzymes were prepared by differential centrifugation as described in the following. Several (15–36) Petri dishes of diameter 15 cm with cells grown to confluence were rinsed twice with 5 mL phosphate-buffered saline (“PBS”: 138 mM NaCl (VWR, Leuven, Belgium), 1.5 mM KH_2_PO_4_ (Sigma-Aldrich, Saint Louis, MO, USA), 8.1 mM Na_2_HPO_4_ (Sigma-Aldrich, Saint Louis, MO, USA), 2.7 mM KCl (MERCK, Darmstadt, Germany), pH 7.4) at room temperature. The cells were harvested at room temperature using a rubber policeman 4 min after addition of 2.8 mL 5 mM EDTA (Sigma-Aldrich, Saint Louis, MO, USA) in PBS to each Petri dish. Cells from 3 Petri dishes were combined, centrifuged 5 min at 2000× *g*, and then resuspended in 4 mL of PBS at 4 °C, followed by centrifugation again for 5 min at 2000× *g*. The cells were resuspended and lysed in 4 mL of a hypotonic solution of 1 mM NaHCO_3_ (Sigma-Aldrich, Saint Louis, MO, USA), pH 7.8, 2 mM CaCl_2_ (Sigma-Aldrich, Saint Louis, MO, USA), 5 mM MgCl_2_ (MERCK, Darmstadt, Germany) (“lysis buffer”) at 4 °C for 10 min and then homogenized with 40 strokes in a 15 mL glass Dounce homogenizer. After centrifugation for 5 min at 3000× *g* at 4 °C, the supernatant was isolated and placed on ice, while 4 mL of the lysis buffer was added to the pellet, which still contained some intact cells as well as cell debris and nuclei. The pellet was resuspended, re-homogenized and centrifuged as described above, except that the centrifugation was carried out at 1500× *g*. The two supernatants were combined and stirred for 10 min at 4 °C with an equal volume of a solution of 2 M NaI (Sigma-Aldrich, Saint Louis, MO, USA), 20 mM EDTA (Sigma-Aldrich, Saint Louis, MO, USA), 5 mM MgCl_2_ (MERCK, Darmstadt, Germany) and 160 mM Tris (Sigma-Aldrich, Saint Louis, MO, USA), pH 8.3, followed by dilution 1:1 with TE buffer (10 mM Tris, pH 7.4, 1 mM EDTA without Na^+^). The plasma membranes were then pelleted by centrifugation at 48,000× *g* for 45 min at 4 °C and subsequently washed with 15 mL TE buffer (without Na^+^) followed by centrifugation at 48,000× *g* for 45 min at 4 °C. When the supernatant was discarded, the mouth and sides of the tube were wiped with a Kleenex hand towel to reduce contamination with Na^+^ and K^+^. Finally, the pellet was resuspended at 4 °C in 100–500 µL TE buffer (without Na^+^) and homogenized with 10 strokes in a small (1 mL) glass homogenizer. Protein concentration was determined by the dye binding method of Bradford [51] using bovine serum albumin as a standard. The protein was then stored in Eppendorf tubes at −80 °C.

To allow access of ATP, Mg^2+^, Na^+^, and K^+^ from both sides of the membrane, the plasma membrane vesicles were made leaky by incubation for 30 min at room temperature with sodium deoxycholate (MERCK, Darmstadt, Germany) 0.65 mg/mL at a protein concentration of 0.4 mg/mL in medium containing in addition 20 mM imidazole (MERCK, Darmstadt, Germany) and 2 mM EDTA (Sigma-Aldrich, Saint Louis, MO, USA) at pH 7.0.

The concentration of Na^+^ in the preparation was determined using a Radiometer FLM3 flame photometer (Radiometer Medical, Copenhagen, Denmark) and this contribution of Na^+^ (less than 100 μM) was taken into consideration when calculating the Na^+^ concentrations in the media in the phosphorylation experiments.

### 4.3. Phosphorylation from [γ-^32^P]ATP

To evaluate the affinity for Na^+^ at the cytoplasmic-facing sites of the expressed mutant and wild-type Na^+^,K^+^-ATPases, the Na^+^ dependence of phosphorylation from ATP (reactions 2 and 3 in Figure 1B) was determined according to previously established principles [35,36]. The leaky membranes were incubated for 10 s at 0 °C in a total volume of 100 μL containing 10 μg membrane protein, 2 μM [γ-^32^P]ATP (3000 Ci/mmol, Revvity, Boston, MA, USA, obtained via Lier, Belgium, mixed with non-radioactive ATP (MERCK, Darmstadt, Germany) to give a final activity of approximately 0.12 MBq/mL with ATP concentration 2 μM), 20 mM Tris (Sigma-Aldrich, Saint Louis, MO, USA), pH 7.5, 3 mM MgCl_2_ (MERCK, Darmstadt, Germany), 1 mM EGTA (Sigma-Aldrich, Steinheim, Germany), 20 μg oligomycin/mL (Sigma-Aldrich, Schelldorf, Germany), 10 μM ouabain (Sigma-Aldrich, Darmstadt, Germany) (inhibiting the endogenous COS-1 cell Na^+^,K^+^-ATPase) and varying concentrations of NaCl (VWR, Leuven, Belgium) and N-methyl-D-glucamine (FLUKA, Steinheim, Germany), the latter varied to maintain the ionic strength constant. The presence of oligomycin slows reaction 4 in Figure 1B [3]. The absence of K^+^ stabilizes the phosphoenzyme further by slowing reactions 5 and 6 in Figure 1B, thereby ensuring maximal accumulation of the phosphoenzyme at the highest Na^+^ concentrations, and moreover prevents competition between Na^+^ and K^+^ at the cytoplasmic-facing sites (reactions 1 and 2 in Figure 1B). Hence, it can be excluded that the apparent affinity for Na^+^ depends on the affinity for K^+^ at these sites. For background subtraction, the phosphorylation experiments were performed in the presence of a high K^+^ concentration of 50 mM KCl (MERCK, Darmstadt, Germany) without added Na^+^, thereby maintaining the enzyme in the non-phosphorylatable E_2_(K_2_) state (Figure 1B).

The formation of phosphoenzyme was terminated after 10 s incubation with [γ-^32^P]ATP by quenching with 500 μL ice-cold 1 M phosphoric acid (MERCK, Darmstadt, Germany) with a pH of 2.4 that stabilizes the aspartyl phosphate bond at the active site. The acid-precipitated ^32^P-labeled phosphoenzyme was washed by centrifugation prior to separation by SDS (MERCK, Darmstadt, Germany) polyacrylamide gel electrophoresis at pH 6.0 for 15 min at room temperature, followed by approximately 1 h at 15 °C (to prevent any degradation of the phosphoenzyme due to heat development). The radioactivity associated with the separated ^32^P-labeled phosphoenzyme band was quantified by phosphor imaging using a Cyclone Plus Storage System (Model C431200, PerkinElmer, Waltham, MA, USA).

### 4.4. ATPase Activity and Catalytic Turnover Rate

Using the colorimetric procedure of Baginski [44,52] to follow the liberation of P_i_, the ATPase activity was determined on the leaky membrane preparation at 37 °C in medium containing the membranes (20 μg membrane protein/mL), 30 mM histidine (Sigma-Aldrich, Darmstadt, Germany), pH 7.4, 130 mM NaCl (VWR, Leuven, Belgium), 20 mM KCl (MERCK, Darmstadt, Germany), 1 mM EGTA (Sigma-Aldrich, Steinheim, Germany), 3 mM MgCl_2_, (MERCK, Darmstadt, Germany), 3 mM ATP (MERCK, Darmstadt, Germany), and 10 μM ouabain (Sigma-Aldrich, Darmstadt, Germany) (inhibiting the endogenous COS-1 cell Na^+^,K^+^-ATPase). In some experiments, the ATP concentration varied between zero and 3 mM with the other components kept at the concentrations indicated above, or the KCl concentration varied between zero and 30 mM at a constant NaCl concentration of 40 mM. For background subtraction, the ouabain concentration was increased to 10 mM to inhibit all Na^+^,K^+^-ATPase activity.

To obtain the catalytic turnover rate for ATPase activity (the number of enzyme cycles per molecule per min), the concentration of active Na^+^,K^+^-ATPase sites was determined by the same phosphorylation protocol as described above with a saturating Na^+^ concentration of 150 mM to obtain stoichiometric phosphorylation. The turnover rate was calculated as the ratio between the ATPase activity and the active site concentration.

### 4.5. Data Analysis and Statistics

Figures and tables show mean values ± standard deviation (SD) (indicated by error bars in the graphs when larger than the size of the symbols). The number of independent experiments is indicated as *n*. For each mutant and wild type, experiments were carried out with different preparations of isolated plasma membranes. The SigmaPlot^®^ software (version 10.0, SYSTAT, Palo Alto, CA, USA) was used for curve fitting by non-linear regression analysis. The Na^+^ concentration dependence of phosphorylation for all mutants and the wild types, the K^+^ concentration dependence of Na^+^,K^+^-ATPase activity for α1-wild type and α1-G321S, and the ATP concentration dependence of Na^+^,K^+^-ATPase activity for α3-wild type and α3-G316S were fitted using a simple Hill function:

(1) $$A=(A_{max}-A_{0})\cdot \left(\frac{{\left[L\right]}^{h}}{K_{0.5}^{h}+{\left[L\right]}^{h}}\right)+A_{0}$$

A represents the phosphorylation or Na^+^,K^+^-ATPase activity level at the actual concentration of activating ligand (L), A_0_ is the value corresponding to a zero ligand concentration (A_0_ = 0 for L being Na^+^ or ATP, but is slightly higher for K^+^), A_max_ is the extrapolated maximal phosphorylation or Na^+^,K^+^-ATPase activity level (set to 100% in the graphs) corresponding to an infinite ligand concentration, K_0.5_ is the ligand concentration giving half-maximum activation (reciprocal of apparent affinity), and h is the Hill coefficient. Mean values of K_0.5_ ± SD were calculated from the individual K_0.5_ values obtained from the independent experiments, each consisting of a series of ligand concentrations. The curves shown in the figures represent the best fits to the complete set of data points from all experiments.

For the α3-wild type and certain mutants the K^+^ concentration dependence of Na^+^,K^+^-ATPase activity is biphasic, showing inhibition at the highest K^+^ concentrations. Curve fitting using SigmaPlot^®^ was in these cases performed using a Hill equation modified by including a negative Hill term to represent the inhibition:

$$A=\frac{A_{max}{\left[L\right]}^{h}}{\left(K_{A}^{h}+{\left[L\right]}^{h}\right)}-\frac{I_{max}{\left[L\right]}^{k}}{\left(K_{I}^{k}+{\left[L\right]}^{k}\right)}+A_{0}$$

The activation and inhibition phases are indexed as A and I, respectively, while h and k are the corresponding Hill coefficients. K_0.5_ values corresponding to the activation phase were read manually as the K^+^ concentration at which the increase in activity is half that corresponding to the peak value of the activity.

Statistical evaluation was performed using the one-way ANOVA for multiple comparisons versus the control group with the Holm–Sidak test.

Structural modeling was performed using PyMOL software (version 0.99, DeLano Scientific LLC, South San Fransisco, CA, USA).

## 5. Conclusion and Perspective

In conclusion, we have found an unexpected increase in the affinity of Na^+^,K^+^-ATPase for Na^+^ caused by the mutation α3-G316S associated with rapid-onset ataxia (ROA). A similar increase was seen for the corresponding α1-G321S mutation. In the neurological disease mutant α3-D923N and the corresponding α1-D928N, which exhibit a strongly reduced Na^+^ affinity due to replacement of an aspartate in Na^+^ site III, introduction of α3-G316S and α1-G321S, respectively, as second-site mutations restores Na^+^ affinity to a wild-type-like level. The glycine is positioned in transmembrane segment M4 (helix M4E) near Na^+^ binding site II. Mutations that break the salt bridge between a glutamate in M4E, two helix-turns below the glycine, closer to the extracellular border, and an arginine in the L6-7 extracellular loop by altering the charge of one of the salt bridge partners likewise rescue Na^+^ affinity in mutants α3-D923N/α1-D928N. Relative to the previously described aspartate replacement of the glutamate in the salt bridge [36], the new salt bridge mutations studied here are super-efficient rescuers and cooperate with the glycine mutation, thereby revealing the molecular mechanism underlying the rescue: the rescue is due to a displacement of the M4E helix toward the cytoplasmic side, similar to the rearrangement occurring during the normal Na^+^,K^+^-ATPase transport cycle as part of the E_2_-to-E_1_ conformational transition, which promotes Na^+^ binding at site II and, thus, phosphorylation from ATP, thereby increasing the apparent Na^+^ affinity and the transport rate.

The perspective of these findings is that the insight provided here might be of value in the development of efficient medications to treat patients with Na^+^,K^+^-ATPase disease mutations that reduce the affinity for Na^+^. The salt bridge is located at the entry of an extracellular-facing cavity between transmembrane helices M2, M4E, M5 and M6 (cf. Figure 1C,D) where the transported ions are tunneled to and from the ion-binding sites and where ouabain and other cardiotonic steroid inhibitors bind from the extracellular side in the E_2_P state. Compounds preventing formation of the salt bridge by binding in this cavity without having the stalling effect on the enzyme cycle seen for several cardiotonic steroids should therefore be in focus in the search for an efficient pharmaceutical. In this context, a limitation associated with the present study is the question of whether the rescue effects observed using the COS-1 expression system can be extrapolated to native neuronal cells under conditions of increased neuronal activity or cellular stress relevant to neurological disorders.

## Figures and Tables

**Table 1:** Expression level of α1- and α3-wild types and mutants.

Protein	Expression Level (pmol/mg Total Membrane Protein) ^1^	% of Wild Type
α1		
α1 wild type	42.78 ± 10.20 (*n* = 10)	100 ± 24
α1-E314D	60.06 ± 28.65 (*n* = 6)	140 ± 67
α1-G321S	91.37 ± 27.98 (*n* = 3) *	214 ± 65
α1-D928N	79.16 ± 25.26 (*n* = 3)	185 ± 59
α1-E314D-D928N	41.78 ± 2.09 (*n* = 4)	98 ± 5
α1-G321S-D928N	72.04 ± 36.79 (*n* = 3)	168 ± 86
α1-E314D-G321S-D928N	51.32 ± 5.18 (*n* = 4)	120 ± 12
α1-E314N	35.91 ± 11.50 (*n* = 3)	84 ± 27
α1-E314R	47.42 ± 10.52 (*n* = 5)	111 ± 25
α1-E314N-D928N	54.54 ± 45.51 (*n* = 4)	127 ± 106
α1-E314R-D928N	36.32 ± 22.31 (*n* = 4)	85 ± 52
α1-R882A	49.11 ± 32.92 (*n* = 4)	115 ± 77
α1-R882A-D928N	17.21 ± 4.00 (*n* = 3)	40 ± 9
α3		
α3-wild type	46.51 ± 13.47 (*n* = 8)	100 ± 29
α3-E309D	41.15 ± 10.24 (*n* = 6)	88 ± 22
α3-G316S	115.64 ± 31.76 (*n* = 4) *	249 ± 68
α3-D923N	55.29 ± 20.73 (*n* = 4)	119 ± 45
α3-E309D-D923N	161.09 ± 42.43 (*n* = 4) *	346 ± 68
α3-G316S-D923N	36.92 ± 13.08 (*n* = 4)	79 ± 28
α3-E309D-G316S-D923N	124.85 ± 48.03 (*n* = 4) *	268 ± 103
α3-E309N	30.80 ± 10.38 (*n* = 6)	66 ± 22
α3-E309R	43.72 ± 4.75 (*n* = 6)	94 ± 10
α3-E309N-D923N	73.91 ± 32.89 (*n* = 6)	159 ± 71
α3-E309R-D923N	32.96 ± 20.96 (*n* = 5)	71 ± 45
α3-R877A	13.56 ± 3.39 (*n* = 5)	29 ± 7
α3-R877A-D923N	40.22 ± 12.74 (*n* = 7)	86 ± 27

^1^ The expression level was determined as the concentration of active Na^+^,K^+^-ATPase sites by phosphorylation capacity. All values are shown with indication of standard deviation (SD) by ± and number of independent experiments by *n*. Values that differ significantly from the corresponding wild type at *p* < 0.05 level in one-way Anova test are marked with *.

**Table 2:** Na^+^ dependence and catalytic turnover rate of α1- and α3-wild types and mutants.

Protein	Na^+^ Dependence, K_0.5_Na^+^ (mM) ^1^	Catalytic Turnover Rate (min^−1^) ^1^, % of Wild Type
α1		
α1-wild type ^2^	0.469 ± 0.067 (*n* = 7) #	8305 ± 973 (*n* = 16), 100% #
α1-E314D ^2^	0.152 ± 0.030 (*n* = 5) #	7732 ± 820 (*n* = 8), 93% #
α1-G321S	0.119 ± 0.016 (*n* = 4) #	6195 ± 366 (*n* = 4), 75% *#
α1-D928N ^2^	38.296 ± 8.550 (*n* = 9) *	3345 ± 599 (*n* = 14), 40% *
α1-E314D-D928N ^2^	4.038 ± 0.695 (*n* = 4) #	5692 ± 1126 (*n* = 8), 69% *#
α1-G321S-D928N	0.533 ± 0.030 (*n* = 3) #	4842 ± 310 (*n* = 6), 58% *#
α1-E314D-G321S-D928N	0.082 ± 0.032 (*n* = 6) #	6092 ± 1059 (*n* = 6), 73% *#
α1-E314N	0.170 ± 0.023 (*n* = 4) #	5144 ± 936 (*n* = 10), 62% *#
α1-E314R	0.141 ± 0.027 (*n* = 4) #	5826 ± 961 (*n* = 10), 70% *#
α1-E314N-D928N	1.399 ± 0.136 (*n* = 4) #	5161 ± 489 (*n* = 5), 62% *#
α1-E314R-D928N	0.620 ± 0.087 (*n* = 3) #	4997 ± 497 (*n* = 5), 60% *#
α1-R882A	0.214 ± 0.020 (*n* = 3) #	2188 ± 283 (*n* = 8), 26% *#
α1-R882A-D928N	1.656 ± 0.685 (*n* = 6) #	3265 ± 356 (*n* = 7), 39% *
α3		
α3-wild type ^2^	0.916 ± 0.105 (*n* = 9) #	7500 ± 661 (*n* = 20), 100% #
α3-E309D	0.353 ± 0.063 (*n* = 7) #	6581 ± 428 (*n* = 4), 88% #
α3-G316S	0.141 ± 0.019 (*n* = 4) #	5215 ± 637 (*n* = 4), 70% *#
α3-D923N ^2^	124.833 ± 15.892 (*n* = 6) *	842 ± 84 (*n* = 10), 11% *
α3-E309D-D923N	40.447 ± 4.076 (*n* = 4) *#	1721 ± 161 (*n* = 8), 23% *#
α3-G316S-D923N	2.073 ± 0.312 (*n* = 3) #	2179 ± 356 (*n* = 10), 29% *#
α3-E309D-G316S-D923N	1.105 ± 0.408 (*n* = 5) #	3293 ± 582 (*n* = 10), 44% *#
α3-E309N	0.305 ± 0.055 (*n* = 4) #	8183 ± 1110 (*n* = 8), 109% #
α3-E309R	0.176 ± 0.033 (*n* = 4) #	7782 ± 1088 (*n* = 6), 104% #
α3-E309N-D923N	7.533 ± 2.405 (*n* = 6) #	2736 ± 699 (*n* = 6), 36% *#
α3-E309R-D923N	3.631 ± 1.588 (*n* = 5) #	2987 ± 1189 (*n* = 9), 40% *#
α3-R877A	0.446 ± 0.049 (*n* = 4) #	5329 ± 878 (*n* = 16), 71% *#
α3-R877A-D923N	31.002 ± 14.192 (*n* = 8) *#	1053 ± 248 (*n* = 6), 14% *

^1^ These values are also presented as bar charts in the figures. All values are shown with indication of standard deviation (SD) by ± and number of independent experiments by *n*. Values that differ significantly from the corresponding wild type at *p* < 0.05 level in one-way Anova test are marked with *. Values that differ significantly from the corresponding disease mutant (α1-D928N or α3-D923N) at *p* < 0.05 level in one-way Anova test are marked with #. ^2^ These parameters have been previously reported [36], however, with slight deviations from the present values, which are based on more independent determinations.

## Data Availability

The original contributions presented in this study are included in the article. Further inquiries can be directed to the corresponding authors.
